# Obligation Is Not a Compulsion—The Quality of the Law and the Effectiveness and Safety of Vaccination against COVID-19

**DOI:** 10.3390/ijerph192114003

**Published:** 2022-10-27

**Authors:** Kamila Kocańda, Dorota Zarębska-Michaluk

**Affiliations:** Collegium Medicum, Jan Kochanowski University of Kielce, IX Wieków Kielc 19A, 25-317 Kielce, Poland

**Keywords:** obligation, compulsion, vaccination against COVID-19, law quality

## Abstract

In December 2021, the Minister of Health in Poland announced via Twitter that vaccination was not compulsory. Such a message from a public authority, who was to a significant extent responsible for organising the process of preventing and combating the infections caused by the SARS-CoV-2 pandemic, appeared to have a negative impact on the public perception of the role of vaccination in combating this disease. The impossibility of directly enforcing vaccination, in the sense that there is no legal basis for its compulsory administration, should not weaken the sense of obligation towards a socially necessary attitude of vaccination as a means of protecting the population against the disease; this should be promoted by public authorities. An auxiliary role in shaping this type of message should be played by the law of appropriate quality, regulating the rules related to vaccination in a way that encourages citizens’ trust in the state and the law.

## 1. Introduction

The only compulsory vaccination in Poland was vaccination against smallpox during the epidemic in 1963 [1,2]. The European Court of Human Rights, when recognising a complaint in 2021 concerning the objection to the statutory obligation to vaccinate children, examined the legality of the solution according to which, in the event of the failure to vaccinate a child, a parent may be fined, and unvaccinated children are not admitted to kindergartens. The complainants saw a violation of the rights included in the Convention for the Protection of Human Rights and Fundamental Freedoms, but the Court ruled that there was no violation. At the same time, the Court pointed out that in the analysed model of the state, compulsory vaccination was not regulated as an absolute obligation, as it could not be directly enforced, and there was no provision allowing for its compulsory administration [3]. During the influenza A (H1N1) pandemic in 2009, the negative consequences of the misconceptions about vaccination were pointed out in a global pandemic scenario [4,5]. A decade later, this risk has reemerged. The issue of vaccination as a source of widespread tension and social divisions within and between communities around the world seems to be particularly important now [6]. Science points to the need for health education and a communication strategy to achieve the acceptance of vaccines on a large scale [7].

## 2. The Quality of the Law concerning the Vaccination against COVID-19 in Poland

At the beginning of 2020, the SARS-CoV-2 coronavirus infection in Poland was covered by the regulations on preventing and combating infections and infectious diseases in humans [8]. On 11 December 2020, a provision was introduced according to which preventive vaccinations against COVID-19 could be carried out by people with a license to practice as a physician, medical assistant [9], nurse, midwife, or work as a paramedic and, under certain conditions, as a school hygienist [10]. On the same day, a team responsible for organising the vaccination of population [11] was appointed, and four days later, a team responsible for the vaccination of the personnel of medical entities was appointed [12]. Initially, vaccination against COVID-19 in Poland had no normative basis because the order of its implementation was specified in the National Vaccination Programme, which had the rank of a Council of Ministers’ resolution, i.e., an internal document [13]. On 31 December 2020, a method of preventing COVID-19 was officially introduced into the legal order, consisting of performing preventive vaccinations against this infectious disease for specific people, using COVID-19 vaccines [14], but it was not until mid-January that the order of vaccination was sanctioned in the rank of an ordinance of the Minister of Health [15]. Months of waiting for the appearance of vaccines did not lead to the proper preparation of the relevant laws. Already at that stage, it was questionable to define the group of persons entitled to vaccination in the regulation, which, despite its solemn name, referring to the national Vaccination Programme, was not universally binding.

The legal regulation on the vaccination against COVID-19 in the rank of an act, contrary to the generally applicable principle of the nonretroactive application of law (lex retro non agit), was implemented in January 2021 with retroactive effect from 27 December 2020 [16]. It predicted that in an epidemic state, vaccination against COVID-19 would be preceded by a pre-vaccination medical examination, and the vaccination process itself was entrusted to physicians, dentists, nurses, midwives, paramedics, and medical assistants. After completion of theoretical and practical training, physiotherapists, pharmacists, school hygienists, and laboratory diagnosticians could also provide vaccinations [17,18]. In April 2021, the number of people authorised to conduct a pre-vaccination medical examination was expanded. Apart from physicians, the group of professions entitled to perform such examinations consisted of dentists, nurses, midwives, medical assistants, paramedics, and school hygienists. After completing theoretical training, the group could be joined by physiotherapists, pharmacists, and laboratory diagnosticians. Under the supervision of a person authorised to perform vaccinations, vaccinations could also be carried out by university students in the fifth year of medicine and third year of nursing. From July 2021, they could also be performed by university students in the fifth year of dentistry, as well as by graduates of these three faculties for six months after graduation [18,19]. In December 2021, it was decided that all persons authorised to perform pre-vaccination medical examinations, other than physicians, could not conduct such examinations for a person under the age of 15 [20]. The wide circle of people authorised by the legislator both to vaccinate and to qualify for vaccination was justified by the initially high demand for people to perform this medical procedure. However, it was difficult to assess the substantive preparation of people who were somehow ad hoc dedicated to its implementation, while normally, in “peacetime”, vaccinations were reserved for primary health care, and it was not ensured in advance that the process of preparing other medical professions for these activities would last longer than a few or several weeks.

Since 1 March 2022, obligatory vaccination against COVID-19 has been introduced in Poland for all persons practising the medical professions in entities conducting medical activities [21]. This obligation applies to physicians, nurses, and medical technicians, as well as technical, administrative, and cleaning personnel employed in a medical entity, i.e., performing professional activities in this entity. The ordinance only indicates that people who have contraindications for vaccination in terms of their health conditions are exempt from the obligation to be vaccinated against COVID-19. The introduced provisions do not contain any sanctions for failure to comply with the vaccination obligation. As underlined by the Ministry of Health, it should be up to an employer to decide how to deal with unvaccinated medical personnel. There are also no provisions on the obligation to provide the employer with a medical certificate stating the existence of contraindications to being vaccinated. In this case, it is up to the employer to decide whether it is sufficient to submit a declaration or whether an appropriate medical certificate given by an employee should be required [22]. Only in the legislative phase was there a draft act aimed at explicitly allowing employers to verify both vaccinations and possible COVID-19 tests. The adopted solutions will determine what procedures are applied in this area, from an employee’s declaration to an obligation to present a vaccination certificate and to attaching this certificate to personal files. Meanwhile, the only sanction is in the Act on Preventing and Combating Infections and Infectious Diseases in Humans, according to which anyone who, in an epidemic state, does not comply with the obligation to carry out preventive vaccinations, set in the Minister of Health’s ordinance, is subject to a financial penalty [23]. Employers do not know how to deal with people for whom a vaccination obligation has been imposed, as it is not compulsory. The legislator seems to assign to citizens, who are individual or institutional employers, the burden of enforcing social responsibility in relation to the order, which has a legal dimension that is not absolute. Even in the face of the fact that obligations are not compulsions, it seems justified to clearly define a type of instrument that may be used in legal transactions, so as not to leave any room for discussion of what is and what is not allowed in the light of an obligation that is not compulsory but has a legal dimension.

## 3. Discussion

Polish law distinguishes between three groups of vaccinations: compulsory for everybody, compulsory for certain professional groups, and recommended [24,25]. In Poland preventive vaccinations are compulsory for the following infectious diseases: diphtheria; tuberculosis; Haemophilus influenzae type b invasive infection; invasive Streptococcus pneumoniae infections; whooping cough; parotid salivary gland inflammation (mumps); measles; chickenpox; acute flaccid childhood paralysis (poliomyelitis); rubella; tetanus; hepatitis B; rabies; and rotavirus infections [26]. It is not necessary to issue a separate administrative decision or other ruling of an imposing nature to implement the vaccination obligation, as it results directly from these provisions, i.e., from the law [27]. An authority competent to carry out the enforcement of non-pecuniary obligations, resulting both from individual decisions issued by it (decisions, rulings) and non-pecuniary obligations arising directly from the generally applicable provisions, is entitled to enforce the vaccination obligation in an administrative manner against a person evading it, which is also an offence, and may impose a fine [28].

In a relevant ordinance, the legislator introduced limitations on the number of people staying in premises, buildings, and facilities; in a specific area of premises, buildings, or facilities; or in open spaces; and participating in assemblies, while providing that people vaccinated against COVID-19 would not be counted if they presented the EU digital COVID-19 certificate or a vaccination certificate. Therefore, no mechanisms for verifying the fact of vaccination have been created, so that entities obliged by the law, under the threat of an administrative penalty in the event of non-compliance with the rules set out in an ordinance, were not able to effectively monitor the number of people subject to the limitations, because they lack the entitlement to control the vaccination status of persons staying in the premises. They are not entitled to request citizens to disclose information on vaccination against COVID-19, as these are sensitive data on health, and only a voluntary decision may be the basis for disclosing these data [29].

Against a person who does not undergo compulsory vaccination, sanitary and epidemiological tests, sanitary procedures, compulsory quarantine or isolation, or hospitalisation, and who is suspected or diagnosed with a particularly dangerous and highly contagious disease that poses a direct threat to the health or life of other people, a direct coercive measure may be used. This consists of holding, immobilising, or forcibly administering drugs [30]. Nevertheless, this provision does not apply to vaccination against COVID-19, not only in relation to the general population but also to the personnel of medical entities who are obligated to undergo vaccination. Obligation is the necessity to do something [31]. Compulsion, on the other hand, is defined as a legal measure compelling one to comply with a legal provision or a court judgement [32]. In a situation where an obligation cannot be effectively enforced, even without a compulsion, due to the lack of instruments to verify its implementation, it becomes a fiction.

Already at the stage of introducing the criteria for the order of vaccination against COVID-19, there was no legal basis for their implementation in a specific scheme, as the National Vaccination Programme was not a source of universally binding law. Then, as also pointed out by the Commissioner for Human Rights, there were no grounds to implement this order by means of an ordinance, because the act provided such an option only in the case of compulsory vaccinations. Vaccination against COVID-19 is not compulsory for the population, although it became compulsory for the personnel of medical entities on 1 March 2022 [33]. The available data show that in recent years the number of cases of evading compulsory vaccinations has increased significantly in Poland. In 2010, there were 3437 cases, and in 2020—over 50,000 cases [34]. According to some authors, the attempt to institutionalise and politicise anti-vaccine activists is part of the wider phenomenon of political populism in the Eastern Europe; therefore, an extensive educational campaign and bottom-up social legal and political measures should be implemented to combat vaccine disinformation [35]. The Polish jurisdiction shows that the relationship between preventive vaccinations and the protection of public health is obvious. First of all, people exposed to the spread of infectious diseases should be protected in this way [36]. The constitutional right to decide about one’s personal life is not absolute and is subject to appropriate limitations, including for health protection [37].

Due to occurring cases of aggression toward personnel performing vaccinations, wider legal protection of persons carrying out the abovementioned medical procedure has been introduced. Whoever infringes the bodily integrity of a person conducting a pre-vaccination medical examination or vaccination against COVID-19 or a person assisting in carrying out the examination or vaccination, who is not entitled to legal protection due to a public official, during or in connection with the examination or vaccination, shall be subject to a fine, the penalty of restriction of liberty, or imprisonment for up to 3 years [38]. At the same time, due to social concerns regarding the safety of vaccines, especially due to their relatively faster introduction to the market, the Protective Vaccine Compensation Fund was established. As indicated in the justification for the act, by which the regulation was implemented into the legal order, the proposed provisions introducing compensation benefits and creating the Fund constitute the implementation of the principle of social solidarity, which is the key to the universal implementation of preventive vaccinations. If, as a result of protective vaccination, a person who has been vaccinated has experienced side effects listed in the Summary of Product Characteristics of the administered vaccine or vaccines, which caused hospitalisation for a period of not less than 14 days or caused an anaphylactic shock requiring the necessity of observation at a hospital emergency department or emergency room or hospitalisation for up to 14 days, he or she will be entitled to a compensation benefit [39].

## 4. Conclusions

The subject-matter literature indicates that the discovery of a vaccine is only the first step [40]. Without prejudging the advantages and efficacy of vaccines from a medical point of view, it should be pointed out that when public authorities recognize the epidemiological legitimacy of the use of certain vaccines, the way in which legislation is made and enforced is essential to achieve the goal of preventing and combating the infections or the diseases they concern. Despite the fact that the legal standards for legalizing the use of certain substances may differ in different countries, and that the procedure of admitting a given vaccine to the market may be adjusted considering the urgency of its use, the decision to use this form of preventing and combating infections is an expression of the pro-health activities of public authorities, which should be consistent at every stage of its implementation. If the safety of each vaccine is the responsibility of the manufacturer, the responsibility for public safety as a result of its common and consistent use is borne by the legislators of a given country, who considered it to be in line with current medical knowledge and an epidemiologically justified method of preventing and combating infections.

Providing all people in need with vaccines [41] should be understood not only in terms of the distribution of preparations in poorer states but also in the sense of responsible health policy in more affluent states. Many reports have underlined the importance and role of providing scientifically valid information on vaccines [42]. Following the influenza A (H1N1) pandemic in 2009, the importance of education on vaccine safety and effectiveness in any future pandemic was emphasised [43,44]. Such a moment has just occurred. It seems that it is time to draw further conclusions, because the ongoing pandemic will probably not be the last one. However, it may turn out to be crucial for the development of effective mechanisms to combat this kind of biological threat, not only in the epidemiological dimension but also in the legal one.
